# Investigation of propyl ethanoate + C_6_–C_10_ 1-alkanols: experimental properties, molecular dynamics, and quantum chemical insights

**DOI:** 10.1039/d6ra01677d

**Published:** 2026-04-29

**Authors:** Mohammad Almasi, Morteza Vatanparast, Adel Noubigh

**Affiliations:** a Department of Applied Chemistry, Faculty of Science, Malayer University Malayer 65174 Iran; b Center for Scientific Research and Entrepreneurship, Northern Border University 73213 Arar Saudi Arabia almasi.mohammad@gmail.com

## Abstract

Accurate knowledge of thermophysical properties and their molecular structural origins is essential for understanding liquid organization in associating mixtures. In this work, the density and viscosity of propyl ethanoate (PE) + C_6_–C_10_ 1-alkanol mixtures were measured over the full composition range at several temperatures, and the corresponding excess molar volumes (*V*^E^) and viscosity deviations (Δ*η*) were determined. To elucidate the structural origins of this behavior, molecular dynamics (MD) simulations and density functional theory (DFT) calculations were combined with experimental observations. Radial and spatial distribution functions reveal that alcohol–alcohol self-association dominates through directional hydrogen bonding, forming transient hydrogen-bonded clusters with dual-donor/acceptor configurations. In contrast, alcohol–ester hydrogen bonding is highly site-specific, occurring exclusively at the ester carbonyl oxygen with significantly weaker intensity. Atoms-in-molecules (AIM) analysis quantifies this interaction hierarchy: ROH–ROH binding energies strengthen from −8.24 to −11.31 kcal mol^−1^ with chain length due to cumulative dispersion interactions, while ROH–PE interactions remain invariant at ∼−8.30 kcal mol^−1^. Void space analysis further demonstrates that the disruption of alcohol hydrogen-bond networks by PE creates expanded free volume, with cavity radii distributions shifting toward larger voids as temperature increases. This structural asymmetry provides a quantitative molecular basis for the observed positive excess molar volumes and negative viscosity deviations, establishing a direct link between hydrogen-bond topology, void distributions, and macroscopic thermophysical behavior in ester–alkanol systems.

## Abstract 2

Accurate knowledge of thermophysical properties and their molecular structural origins is essential for understanding liquid organization in associating mixtures.

## Graphical abstract

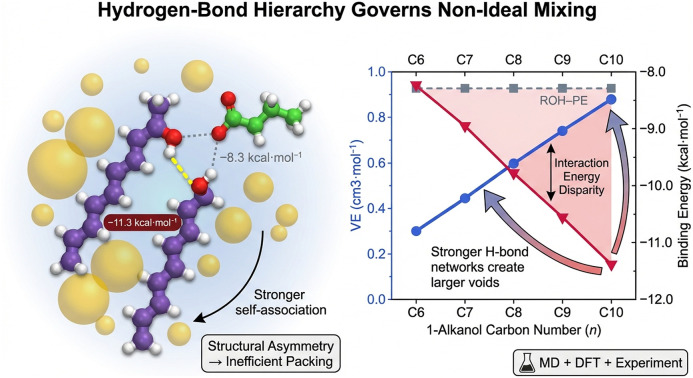

## 1. Introduction

The rational design of chemical processes involving liquid mixtures demands accurate prediction of thermophysical properties—particularly density and viscosity—which govern heat and mass transfer operations, pressure drop calculations, and equipment sizing. These macroscopic transport properties are fundamentally encoded in molecular-scale structure, specifically the topology of hydrogen-bonding networks, packing efficiency, and free volume distributions. Recent advances in multiscale molecular simulation have enabled quantitative relationships between these microscopic descriptors and bulk thermodynamic behavior to be established with unprecedented fidelity. For instance, molecular dynamics simulations of ionic liquid–alcohol mixtures have demonstrated that hydrogen bond network weakening and void space creation directly correlate with thermodynamic deviations, providing a structural basis for non-ideal mixing. Similarly, machine learning approaches combined with molecular simulations have enabled high-throughput screening of thermophysical properties, revealing that hydrogen bond lifetimes serve as reliable predictors for transport behavior in complex fluids. Nevertheless, for ester–alcohol mixtures of industrial relevance—widely employed as solvents, extractants, and fuel additives—a direct and physically transparent bridge between hydrogen-bond topology, void space characteristics, and macroscopic excess properties remains elusive. This gap limits the predictive capability of both equation-of-state frameworks and activity coefficient models for process simulation.^1–4^ Ester–alcohol mixtures play a central role in chemical and energy-related industries, serving as solvents, extractants, fuel components, and formulation media in coatings, pharmaceuticals, flavors, and biodiesel technologies.^5–11^ Among these systems, acetate esters combined with higher alcohols (C_6_–C_10_) are particularly relevant because they offer a balance between solvent power, volatility, and viscosity that is advantageous for process operability and environmental performance. Accurate thermophysical property data for such mixtures are therefore essential for reliable process design, including equipment sizing, flow assurance, heat-transfer calculations, and separation unit optimization.

Previous experimental studies have reported volumetric, calorimetric, and transport properties for various ester–alcohol systems, demonstrating that excess properties are sensitive to both temperature and molecular structure.^5–7^ In general, positive excess molar volumes and non-linear viscosity behavior have been attributed to hydrogen bonding and packing effects. However, most existing studies focus on short-chain alcohols or report macroscopic trends without providing a consistent molecular-level explanation for how alcohol chain length alters the balance between self-association and cross-association in ester–alcohol mixtures. As a result, the microscopic origin of chain-length-dependent excess properties remains insufficiently understood, limiting the transferability of empirical correlations and group-contribution models.

Molecular simulation techniques provide a powerful route to bridge this gap. Classical molecular dynamics (MD) simulations can quantify local liquid structure through radial and spatial distribution functions, hydrogen-bond statistics, and free-volume characteristics, while quantum chemical calculations offer detailed insight into the energetics and directionality of specific intermolecular interactions.^14–16^ Despite their promise, establishing a direct and physically transparent link between microscopic descriptors (*e.g.*, hydrogen-bond topology or void distributions) and macroscopic excess properties such as excess molar volume and viscosity deviations remains challenging. Statistical mechanical formalisms, including Kirkwood–Buff theory, offer a rigorous framework but are often difficult to apply quantitatively to complex associating liquids.^17–19^

In this work, we address these challenges by presenting a comprehensive multiscale study of propyl ethanoate (PE) + C_6_–C_10_ 1-alkanol mixtures. New experimental density and viscosity data were measured over wide composition and temperature ranges, and the corresponding excess molar volumes and viscosity deviations were evaluated and correlated using the Redlich–Kister equation. Furthermore, by employing a combined Molecular Dynamics (MD) and Quantum Chemical (QC) approach, we aim to deliver deep molecular-level insights into the hydrogen bonding networks and dipole–dipole interactions within these mixtures. This comprehensive, multi-scale understanding bridges the critical gap between macroscopic fluid behavior and microscopic interactions, providing the foundational knowledge required to improve predictive thermodynamic models and optimize sustainable, green solvent extraction processes in chemical engineering applications.

## 2. Computational and experimental methods

### 2.1. Molecular dynamics simulations

**Fig. 1:**
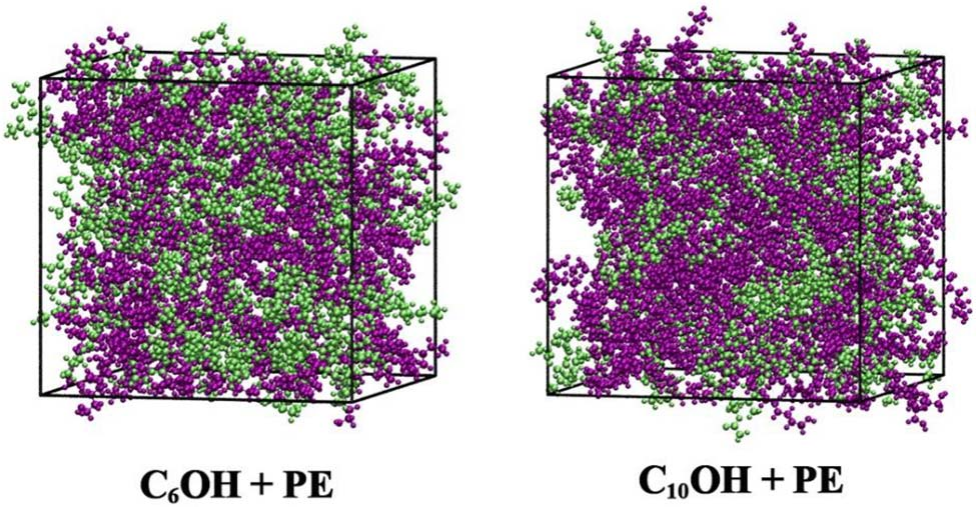
Final snapshot for the system of *x*_1_ = 0.5. ROH-purple, PE-lime.

Molecular dynamics (MD) simulations were performed to gain insight into the microscopic structure and dynamic behavior of the studied mixtures. All simulations were carried out using the GROMACS simulation package.^20^ The OPLS-AA all-atom force field was selected because of its proven reliability in modeling organic liquids, particularly systems containing esters and alcohols.^21–26^ Initial molecular configurations were generated with the PACKMOL program,^26^ by randomly placing 400 molecules in a cubic simulation box with periodic boundary conditions applied in all three spatial directions. The systems were first subjected to energy minimization using the steepest descent method. Subsequently, equilibration was carried out in two stages: (i) a 4 ns simulation in the NVT ensemble controlled by a velocity-rescale thermostat, followed by (ii) a 4 ns NPT simulation employing the C-rescale at a pressure of 1 bar. After equilibration, production simulations were conducted for 10 ns using a time step of 2 fs while maintaining the same thermodynamic conditions. Long-range electrostatic interactions were treated using the particle mesh Ewald (PME) approach, whereas a cutoff distance of 1.2 nm was applied for Lennard–Jones interactions and real-space coulombic terms. All bond lengths were constrained using the LINCS algorithm. Representative snapshots of the equilibrated systems at a PE mole fraction of *x*_1_ = 0.5 are presented in Fig. 1 for the C_6_OH and C_10_OH mixtures.

To ensure the reliability of the simulation results, the OPLS-AA force field was validated against experimental density data for both pure components and their binary mixtures. For the pure liquids at 293.15 K, simulated densities of propyl ethanoate and 1-hexanol showed deviations of less than 1.5% compared to experimental values. To establish transferability to the mixed phase, densities were calculated for mixtures (*x*_PE_ = 0.25, 0.55, and 0.750) across the entire C_6_–C_10_ series. The agreement remained excellent, with deviations of 0.9% for the 1-hexanol system, 1.1% for 1-heptanol, 1.2% for 1-octanol, 0.8% for 1-nonanol, and 1.3% for 1-decanol, confirming the robustness of the force field for these binary mixtures.

While calculation of absolute viscosities from MD was beyond the scope of this study due to the extensive simulation times required for convergence with non-polarizable force fields, the experimental viscosity deviations are interpreted through the structural changes captured in our RDF and void analysis.

### 2.2. Experimental section

Propyl ethanoate (≥99.5% purity) was obtained from Sigma-Aldrich and used without further purification. The 1-alkanols (1-hexanol, 1-heptanol, 1-octanol, 1-nonanol, and 1-decanol) were obtained from Merck with stated purities exceeding 99% by mass fraction. All chemicals were stored over molecular sieves (4 Å) and handled under dry nitrogen to minimize moisture uptake. Data for the pure 1-alkanols were obtained from our previous investigations.^12,13^ Additionally, the experimentally obtained values of density and viscosity for propyl ethanoate, together with those of its binary combinations with 1-alkanols, have been compiled in Table S1.

Density and viscosity measurements were conducted using an Anton Paar SVM 3000 digital rotational viscometer, an instrument designed for characterizing fluids within the low-to-moderate viscosity range. The operational principle of this apparatus is based on rotational rheometry, employing a coaxial dual-cylinder configuration. In this arrangement, the outer cylinder rotates at controlled angular velocities to impose shear deformation on the sample, while the inner cylinder remains fixed in position. The drag forces experienced by the stationary inner cylinder are subsequently converted into torque measurements, enabling quantification of the sample's resistance to flow. This differential rotation mechanism provides enhanced detection capability for variations in viscous behavior across the measured specimens.

Prior to each experimental series, calibration of the viscometer was performed using freshly degassed, double-distilled water to ensure measurement accuracy and reproducibility. Temperature regulation throughout all measurements was achieved *via* the instrument's integrated solid-state thermostat, which maintained thermal stability with an uncertainty not exceeding ±0.02 K. The reliability and accuracy of the experimental setup were further confirmed through a standardization procedure utilizing degassed, deionized water, ensuring conformity with internationally recognized metrological standards.

Regarding sample preparation, all reagent materials underwent degassing treatment prior to measurement. This procedure was implemented to remove dissolved atmospheric gases that could potentially induce bubble nucleation during measurement, thereby compromising the accuracy of rheological determinations. Binary mixtures were prepared using gravimetric methodology with a Mettler AE 163 analytical balance, which provides mass determination with an uncertainty of ±0.14 mg. For each binary system investigated, ten discrete compositions spanning the full mole fraction range were systematically prepared. The uncertainty associated with mixture composition was determined to be ±0.001 in mole fraction units. The expanded uncertainty for density determinations was established at 0.001 g cm^−3^, incorporating contributions arising from both instrumental resolution limitations and thermal equilibration processes. Correspondingly, the relative expanded uncertainty associated with viscosity measurements was determined to be 0.05.

### 2.3. Quantum chemical calculations

To support the experimental results and provide molecular-level understanding of electronic interactions between species, quantum mechanical computations based on density functional theory (DFT) were carried out utilizing the Gaussian 09 software package.^27^ Structural optimization of all molecular geometries was performed employing the M06-2X functional coupled with the 6-311++G** basis set,^28^ a theoretical approach recognized for its accuracy in characterizing weak non-covalent interactions, encompassing hydrogen bond formation and dispersion contributions.

Given the significant conformational freedom of both alcohol molecules and propyl ethanoate, an extensive configurational search was conducted. Multiple starting arrangements were constructed for each pairs, differing in the mutual orientation of hydroxyl and carbonyl groups, the relative positioning of alkyl chains, and internal conformations of the monomers (such as anti and gauche geometries). Each generated structure was independently optimized without constraints. Harmonic vibrational frequency calculations were subsequently performed to confirm that all optimized geometries corresponded to true energy minima. Binding energies were determined according to:*E* = *E*_dimer_ − (*E*_monomer 1_ + *E*_monomer 2_)where *E*_dimer_ denotes the total energy of the optimized complex, and *E*_monomer_ represents the energy of the isolated ROH or PE molecule calculated at the same theoretical level. Basis set superposition error was corrected using the counterpoise approach introduced by Boys and Bernardi,^29,30^ ensuring more accurate estimation of interaction strengths.

The quantum chemical calculations serve a dual purpose in this work: (i) providing fundamental understanding of the interaction hierarchy that governs mixing behavior, and (ii) generating molecular descriptors that could be incorporated into QSPR (Quantitative Structure–Property Relationship) models for property prediction. The binding energies calculated at the M06-2X/6-311++G** level represent benchmark values that can be used to parameterize or validate simplified models (*e.g.*, group contribution methods) employed in process engineering software.

## 3. Results and discussion

### 3.1. Radial distribution function (RDF) analysis

**Fig. 2:**
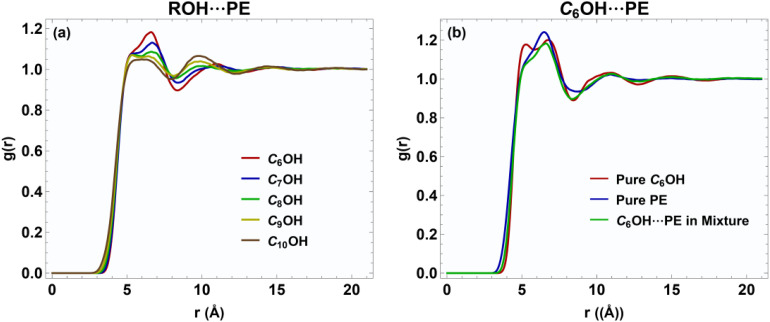
Center of geometry RDF, *g*(*r*), for (a) ROH–PE interactions in equimolar mixtures (*x*_1_ = 0.5) and (b) pure C_6_OH, pure PE, and their equimolar mixture at 293.15 K.

Fig. 2(a) shows the center-of-geometry radial distribution functions (*g*(*r*)) between the alcohol molecules (C_6_OH, C_7_OH, C_8_OH, C_9_OH, and C_10_OH) and propyl ethanoate (PE) at a mole fraction *x*_1_ = 0.5. The RDF between the 1-alkanols and propyl ethanoate shows a pronounced structure in the 5–8 Å region that appears as a split (double) peak rather than a single, sharp maximum. This bimodal feature indicates two preferred center-to-center separations in the first/inner solvation region. Physically, the split peak can be understood as a superposition of distinct relative arrangements of the alcohol and ester molecules: one population in which the alcohols approach PE with their polar (OH) ends relatively close to the ester functional group, and a second population in which the alkyl tails are oriented nearer to the PE center or steric constraints force a larger center-of-geometry separation. Because the plotted RDF is based on molecular centers rather than atom–atom distances, these two orientations (head-on *vs.* tail-dominated approaches) naturally produce two nearby maxima. The systematic decrease of the peak amplitude with increasing alkyl length further supports a steric/packing interpretation: longer chains reduce the probability of very close center-of-geometry approaches, broadening and splitting the first coordination feature.

Fig. 2(b) and S1 show that upon forming the 50 : 50 mixture, the heights of the first-shell peaks are reduced relative to the pure components. This decrease indicates a weakening of short-range positional correlations and less efficient local packing in the mixture. The same qualitative trend is observed for all other alcohols in the C_6_OH–C_10_OH series, demonstrating that the reduction in first-shell ordering upon mixing with propyl ethanoate is a general feature of these systems. This reduction in local packing efficiency is consistent with an excess molar volume *V*^E^ > 0 (positive volume deviation on mixing), because fewer close contacts and increased free volume around molecules will tend to expand the mixture relative to the ideal additivity of pure component volumes.

### 3.2. Spatial distribution function (SDF) analysis

**Fig. 3:**
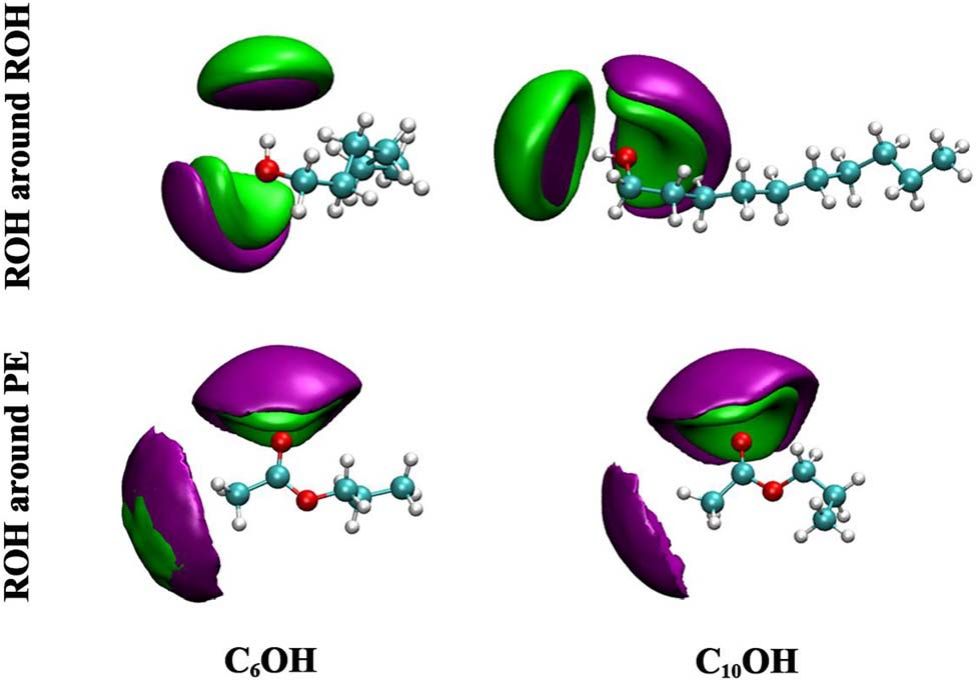
Spatial distribution functions showing the three-dimensional probability density of ROH around ROH and PE in the equimolar mixture (*x*_1_ = 0.5) at 293.15 K, where green lobes correspond to hydrogen atoms and purple regions indicate oxygen atoms.

The spatial distribution functions (SDFs) presented in Fig. 3 and S2 further clarify the microscopic origin of the structural features observed in the RDF analysis. In these representations, the purple isosurfaces correspond to the oxygen atoms of the alcohol molecules, while the green isosurfaces represent the hydrogen atoms of the hydroxyl groups.

RDFs indicates that ROH⋯ROH interactions are dominated by directional hydrogen bonding, and that each alcohol frequently engages in two hydrogen-bond contacts. This is seen in the SDFs as two distinct green (H atom) lobes around the purple (O atom) site, consistent with an alcohol acting both as hydrogen-bond donor and as acceptor. The geometry of these lobes implies the formation of small hydrogen-bonded motifs (for example, dimers, cyclic trimers, or short chains) rather than isolated single H-bonds. Such local bonding motifs lead to transient clusters of hydrogen-bonded alcohols whose size and lifetime depend on chain length and temperature; the presence of two preferred H positions around the alcohol oxygen provides a clear structural basis for cluster formation and the strongly anisotropic ROH–ROH spatial correlations reported above.

In contrast, ROH⋯PE correlations show that hydrogen bonding to the ester is highly site-specific: only the carbonyl oxygen of propyl ethanoate (C=O) is observed to accept hydrogen bonds from the alcohol hydroxyl, while the alkoxy/ester oxygen (the O–C oxygen) shows negligible H-acceptor density in the SDFs. This selective acceptance by the carbonyl oxygen is consistent with its higher electron density and stronger hydrogen-bond basicity, together with steric constraints that disfavor effective approach to the alkoxy oxygen. The net result is a limited number of cross-species hydrogen bonds, whereas ROH⋯ROH hydrogen bonding remains the dominant directional interaction. This strong alcohol self-association combined with only modest cross-association to PE, rationalizes why mixing reduces short-range ordering (lower first-shell *g*(*r*)) and supports the thermodynamic observation of positive excess molar volumes: persistent ROH clusters reduce the propensity for close, efficient packing between unlike molecules.

### 3.3. Combined distribution functions

**Fig. 4:**
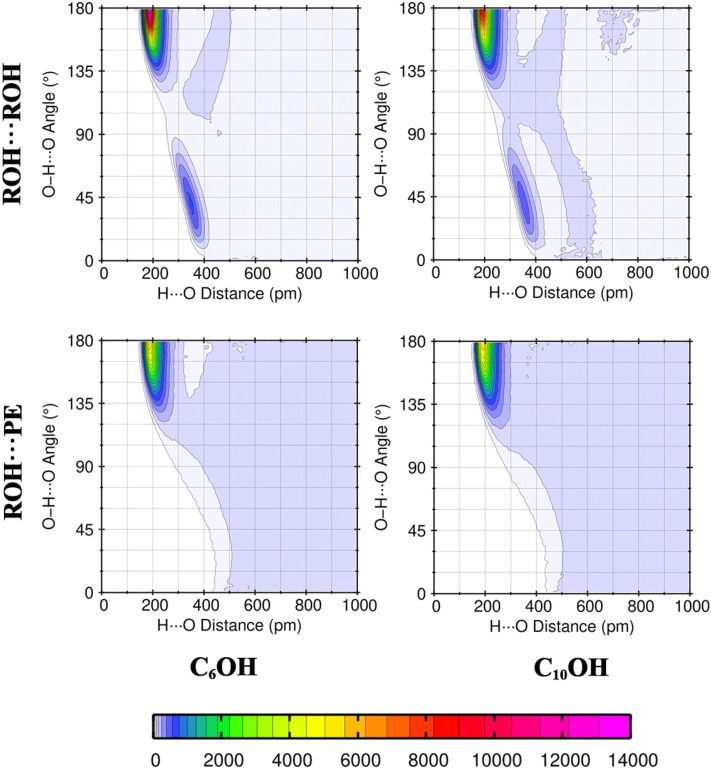
Computed combined distribution functions involving the O–H⋯O hydrogen-bond angle and the H⋯O distance in the equimolar mixture (*x*_1_ = 0.5) at 293.15 K.

Fig. 4 and S3 present combined radial/angular distribution function of the H⋯O distance and the O–H⋯O hydrogen-bond angle for ROH⋯ROH and ROH⋯PE interactions in mixtures. For ROH⋯ROH pairs, two distinct high-probability regions are clearly observed. The first corresponds to short H⋯O distances (≈180–220 pm) and near-linear O–H⋯O angles (≈160–180°), characteristic of strong, well-defined hydrogen bonds. A second, broader population appears at longer distances (≈350–400 pm) and smaller angles (≈15–60°), indicating a secondary hydrogen-bonding configuration in which alcohol molecules participate in additional, weaker or distorted hydrogen bonds. The coexistence of these two populations confirms that alcohol molecules can simultaneously act as hydrogen-bond donors and acceptors, enabling the formation of extended hydrogen-bonded motifs and transient ROH clusters in the liquid phase.

In contrast, the ROH⋯PE distributions exhibit a single dominant hydrogen-bond population centered at short H⋯O distances and near-linear angles, corresponding exclusively to hydrogen bonding between the alcohol hydroxyl hydrogen and the carbonyl oxygen of propyl ethanoate. No secondary hydrogen-bond population is observed for ROH⋯PE interactions, and the ester alkoxy oxygen does not contribute measurably to hydrogen bonding. This highlights the highly selective nature of cross-association, in which only the carbonyl oxygen acts as an effective hydrogen-bond acceptor. Moreover, the intensity of the ROH⋯PE hydrogen-bond population is weaker and more localized than that of ROH⋯ROH, indicating fewer and less persistent cross-species hydrogen bonds. This strong, multiply connected ROH⋯ROH hydrogen bonding *versus* limited ROH⋯PE bonding, explains the persistence of alcohol-rich hydrogen-bonded clusters upon mixing and provides a microscopic structural basis for the reduced local packing efficiency and positive excess molar volumes observed for these systems.

### 3.4. Temperature dependence of RDFs

**Fig. 5:**
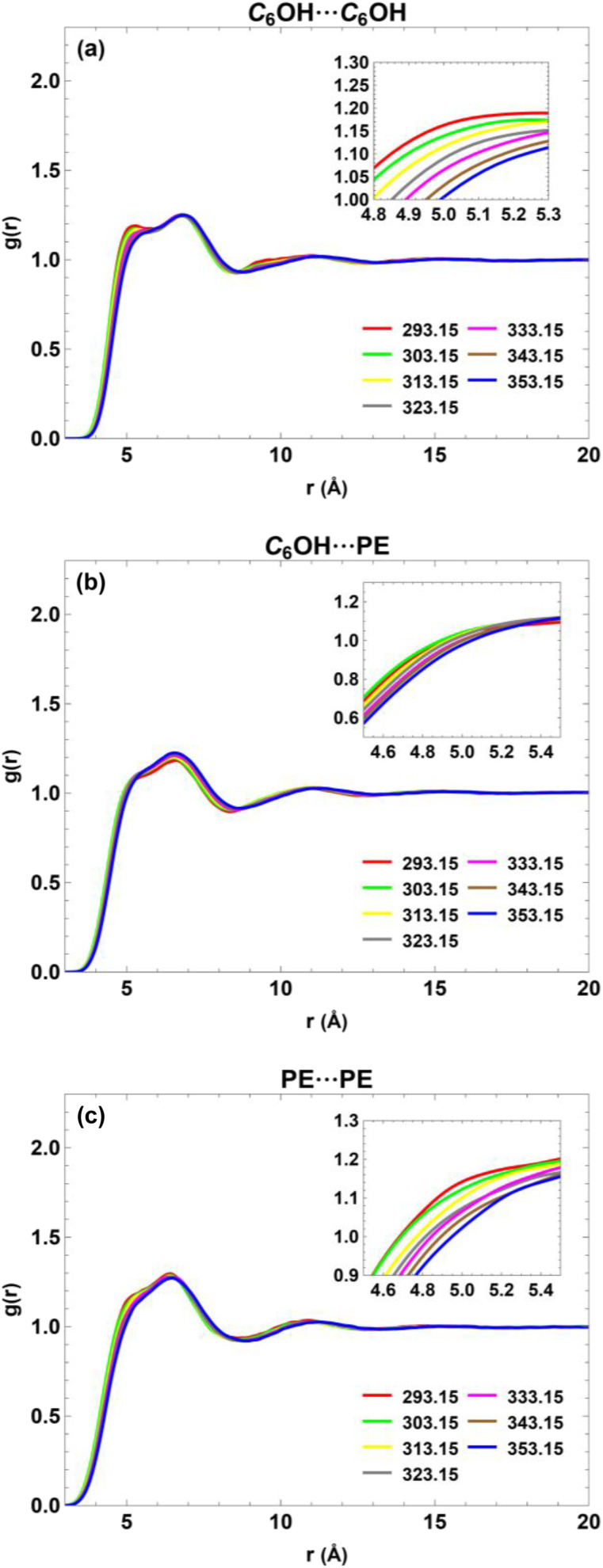
Center-of-geometry RDF profiles for C_6_OH–C_6_OH, C_6_OH–PE, and PE–PE interactions at equimolar composition (*x*_1_ = 0.5) over the temperature range 293.15–353.15 K.

Fig. 5 presents the center-of-geometry radial distribution functions for C_6_OH–C_6_OH, C_6_OH–PE, and PE–PE interactions at equimolar composition (*x*_1_ = 0.5) over the temperature range 293.15–353.15 K. For all interaction pairs, increasing temperature leads not only to a systematic decrease in the height of the first coordination peak but also to a noticeable shift of this peak toward larger intermolecular separations. The displacement of the first maximum to higher *r* reflects thermal expansion of the liquid and an increase in the average nearest-neighbor distance as thermal motion becomes more pronounced.

The temperature-induced shift is most evident for the C_6_OH–C_6_OH interactions. At lower temperatures, the first peak occurs at shorter distances with higher intensity, consistent with compact local structures stabilized by hydrogen bonding. As temperature increases, partial disruption of the hydrogen-bond network leads to both a reduction in peak height and an outward shift of the peak position, indicating looser local packing and longer average hydrogen-bond distances. This behavior supports the presence of temperature-sensitive hydrogen-bonded clusters that expand and weaken upon heating. In contrast, the C_6_OH–PE and PE–PE RDFs show smaller but still discernible shifts of the first coordination peak to higher *r*, accompanied by more moderate reductions in peak height. These trends are characteristic of interactions dominated by dispersive forces and packing effects rather than strong directional bonding. Collectively, the combined reduction in peak intensity and outward shift of the first coordination shell with temperature provides a consistent microscopic explanation for the increase in free volume and the corresponding rise in excess molar volume observed at elevated temperatures.

### 3.5. Void distribution analysis

**Fig. 6:**
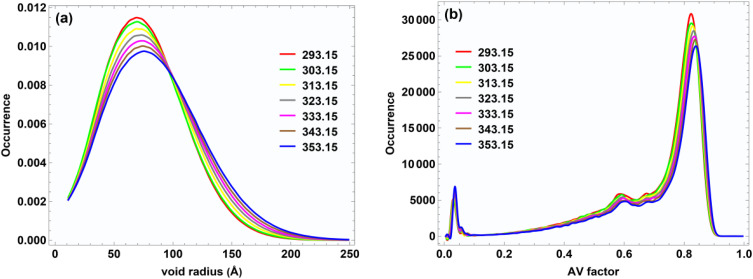
Void analysis in C_6_OH–PE mixtures at *x*_1_ = 0.50: (a) cavity size distribution and (b) isoperimetric ratio (AV factor) distribution.

Fig. 6 presents the analysis of void (cavity) distributions in the equimolar PE + C_6_OH mixture at different temperatures. Fig. 6(a) shows the probability distribution of cavity radius, while Fig. 6(b) illustrates the distribution of the isoperimetric ratio (AV factor).

As shown in Fig. 6(a), the cavity radius distributions exhibit a single broad maximum, indicating the predominance of intermediate-sized voids within the liquid structure. With increasing temperature from 293.15 to 353.15 K, the peak position shifts toward larger cavity radii accompanied by a noticeable broadening of the distribution. This behavior reflects the progressive thermal expansion of the mixture and the weakening of intermolecular interactions, particularly hydrogen bonding between the hydroxyl group of C_6_OH and the carbonyl oxygen of propyl ethanoate. At lower temperatures, the narrower distributions and smaller most probable cavity radii suggest a more compact liquid structure, dominated by stronger hetero-molecular associations. In contrast, elevated temperatures promote increased molecular mobility and disrupt local packing efficiency, resulting in the formation of larger voids and enhanced free volume. This temperature-induced evolution of cavity sizes is consistent with the experimentally observed increase in excess molar volume for mixtures and supports the interpretation of positive deviations from ideal mixing.

Fig. 6(b) depicts the distribution of the isoperimetric ratio (AV factor), which provides insight into the geometrical characteristics of the cavities. The distributions are strongly skewed toward high AV values (≈0.80–0.85), indicating that most cavities retain a relatively compact and near-spherical shape. However, as temperature increases, a slight shift of the distribution toward higher AV values is observed, accompanied by a reduction in peak occurrence. With increasing temperature, a slight shift of the distribution toward higher AV values is observed, accompanied by a reduction in peak occurrence.

Fig. S4 presents molecular dynamics snapshots illustrating the spatial distribution of void spheres with radii of 70 pm and 150 pm in the equimolar propyl ethanoate + C_6_OH system at 293.15 and 352.15 K. The yellow regions represent the identified void space within the liquid matrix. At the smaller probe radius (70 pm), a dense and highly interconnected network of voids is observed at both temperatures, indicating the presence of numerous small cavities arising from local packing inefficiencies between PE and C_6_OH molecules. At 293.15 K, these voids appear relatively uniformly distributed, reflecting a compact liquid structure stabilized by hetero-molecular interactions, particularly hydrogen bonding between the hydroxyl group of C_6_OH and the carbonyl oxygen of PE. Upon increasing the temperature to 352.15 K, the spatial continuity of the 70 pm void network becomes less pronounced, with a noticeable reduction in void density. In contrast, at the larger probe radius (150 pm), significantly fewer voids are detected at 293.15 K, indicating that large cavities are rare in the tightly packed liquid structure. However, at 352.15 K, the number and spatial extent of 150 pm voids increase markedly. This reflects the formation of larger free-volume regions driven by thermal expansion and weakened intermolecular associations.

### 3.6. Density and excess molar volume

**Fig. 7:**
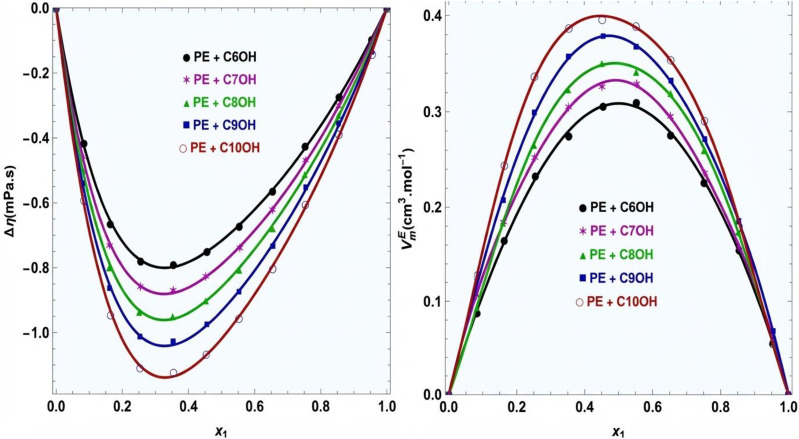
(a) Viscosity deviation Δ*η* and excess molar volumes for PE + 1-alkanols at 293.15 K. (—) Redlich–Kister equation.

Fig. 7(a) shows the excess molar volume *V*^E^ for PE + 1-alkanol mixtures at 293.15 K as a function of mole fraction. For all systems, *V*^E^ is positive over the entire composition range and exhibits a clear maximum near the equimolar region, indicating volume expansion upon mixing. The magnitude of *V*^E^ increases systematically with increasing alkyl chain length of the alcohol, following the order C_6_OH < C_7_OH < C_8_OH < C_9_OH < C_10_OH. This trend reflects progressively less efficient packing as the alcohol chain length increases, consistent with the molecular-level observations from RDF and SDF analyses. Strong ROH–ROH hydrogen bonding leads to self-associated alcohol clusters, while cross-association between ROH and PE is limited and site-specific, occurring primarily *via* the ester carbonyl oxygen. The resulting imbalance between strong self-association and weaker unlike interactions inhibits compact packing in the mixture, generating excess free volume and positive *V*^E^. The smooth curves obtained from the Redlich–Kister equation indicate that the experimental data are well represented by this model.

The positive excess molar volumes observed for all PE + 1-alkanol mixtures have direct implications for process design: 1. storage tank sizing: the volume expansion upon mixing (up to 0.89 cm^3^ mol^−1^ at equimolar composition for PE + 1-decanol) must be accounted for when sizing storage and blending vessels. 2. Metering and flow control: non-ideal density behavior affects mass flow calculations from volumetric measurements. Process control strategies should incorporate composition-dependent density corrections. 3. Heat exchanger design: density variations influence Reynolds numbers and heat transfer coefficients. The correlations developed here enable accurate calculation of these parameters across the composition range. The Redlich–Kister parameters can be directly implemented in process simulation software to compute mixture densities at any composition within the experimental range.

### 3.7. Viscosity and deviations

Fig. 7(b) presents the viscosity deviations Δ*η* for the same systems, which are negative across the entire composition range and show minima near intermediate mole fractions. The increasingly negative magnitude of Δ*η* with alcohol chain length mirrors the behavior observed for *V*^E^, suggesting a common structural origin. Negative viscosity deviations indicate that the mixture flows more easily than predicted from ideal mixing, which can be attributed to the partial disruption of the alcohol hydrogen-bond network upon addition of PE. As PE molecules penetrate the alcohol-rich regions, they weaken ROH–ROH connectivity without forming equally strong cross-species hydrogen bonds, thereby reducing resistance to flow. The combined observation of positive excess molar volumes and negative viscosity deviations provides consistent macroscopic evidence of reduced local ordering and inefficient packing, in agreement with the reduced first-shell RDF intensities and selective hydrogen-bonding patterns identified in the microscopic structural analysis.

### 3.8. Quantum chemical calculations

The interaction energies reported in Table 1 were obtained from binary (dimer) quantum-chemical calculations performed using the Gaussian software package. For each system, a single propyl ethanoate molecule and a single alcohol molecule (or two identical molecules for ROH–ROH and PE–PE) were placed in an initial configuration and fully optimized at the M06-2X/6-311++G** level of theory. The resulting binding energies therefore represent intrinsic pairwise interaction strengths in the gas phase, free from bulk packing effects and many-body contributions. Despite this simplification, the calculated energies provide valuable qualitative insight into the relative strength of self- and cross-association in the liquid mixtures.

**Table 1:** Binding energies (in kcal mol^−1^) at M06-2X/6-311++G** level of theory

System	Binding energies of ROH-ROH (*E*_1_)	Binding energies of ROH-PE (*E*_2_)	Binding energies of PE-PE (*E*_3_)	*E*_2_ − *E*_1_	*E*_2_ − *E*_3_
PE-C_6_OH	−8.237	−8.314	−5.958	−0.077	−2.356
PE-C_7_OH	−8.890	−8.307	−5.958	0.583	−2.349
PE-C_8_OH	−9.719	−8.306	−5.958	1.413	−2.349
PE-C_9_OH	−10.485	−8.303	−5.958	2.182	−2.345
PE-C_10_OH	−11.308	−8.300	−5.958	3.008	−2.342

**Fig. 8:**
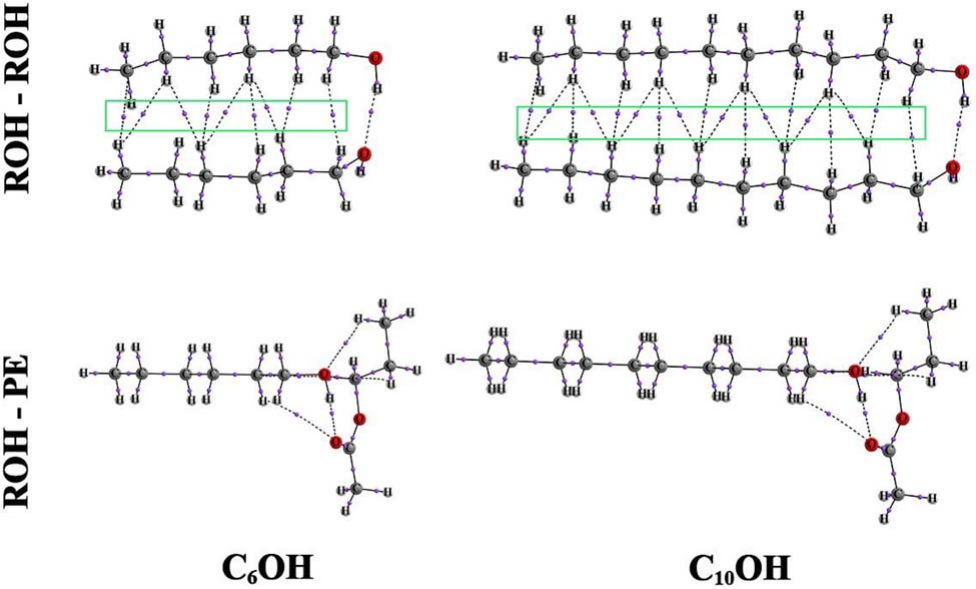
AIM analysis graph showing the presence of bond critical points associated with hydrogen bonding and dispersion (green box) interactions in PE + ROH systems.

In particular, the dimer calculations reveal that ROH–ROH interactions become increasingly stronger than ROH–PE interactions as the alcohol chain length increases, while ROH–PE binding energies remain nearly invariant across the homologous series. This confirms that the dominant contribution to alcohol–ester association arises from a localized hydrogen bond between the alcohol hydroxyl group and the ester carbonyl oxygen, whereas the alkyl chain length primarily enhances alcohol self-association through dispersion interactions. In addition, atoms-in-molecules (AIM) analyses were performed to elucidate the nature of the intermolecular interactions governing the mixing behavior. The AIM results reveal that, as illustrated in Fig. 8, when alcohol molecules are arranged adjacent to one another, bond critical points appear between neighboring alkyl chains, indicating the presence of stabilizing dispersion interactions. As the length of the alcohol alkyl chain increases, the number of these critical points also increases, reflecting the progressive enhancement of dispersion forces with increasing chain size. This strengthening of alcohol–alcohol interactions promotes self-association and contributes to inefficient packing upon mixing with propyl ethanoate, thereby supporting the experimentally observed positive excess molar volumes.

Furthermore, the calculations demonstrate that the interaction between alcohol molecules and propyl ethanoate is highly localized and occurs primarily between the hydrogen atom of the alcohol hydroxyl group and the carbonyl oxygen atom of propyl ethanoate. AIM analysis confirms the presence of a bond critical point associated with this O–H⋯O hydrogen bond, while no such critical points are detected involving the alkoxy oxygen of the ester. The optimized geometries show that increasing the length of the alcohol alkyl chain does not significantly alter either the relative orientation of the interacting functional groups or the strength of this hydrogen bond. This insensitivity to chain length explains the nearly constant ROH–PE binding energies obtained from the dimer calculations and further supports the conclusion that alcohol self-association, rather than cross-association with propyl ethanoate, dominates the microscopic structure and thermodynamic behavior of these mixtures.

While excess molar volume and viscosity are bulk properties governed by collective behavior, quantum chemical calculations provide essential insight into the hierarchy of intermolecular interactions that drive macroscopic non-ideality. The calculated gas-phase binding energies reveal that alcohol–alcohol interactions strengthen significantly with increasing alkyl chain length, whereas alcohol–propyl ethanoate interactions remain nearly invariant across the homologous series.

From a chemical engineering perspective, this interaction hierarchy explains why conventional activity-coefficient models assuming uniform interaction strength may fail for ester–alkanol mixtures. The growing disparity between self- and cross-interactions with chain length leads to increasing deviations from ideality, which must be explicitly captured in thermodynamic models used for phase equilibrium and transport property prediction.

Atoms-in-molecules (AIM) analysis further demonstrates that the enhanced stability of longer-chain alcohol clusters arises not solely from hydrogen bonding but also from cumulative dispersion interactions between alkyl chains. This finding is particularly relevant for the parameterization of group-contribution methods, where dispersion contributions are often underrepresented.

Although the present DFT calculations are limited to dimer interactions and do not capture many-body effects present in the liquid phase, they provide valuable benchmarks for the development and validation of simplified interaction models suitable for incorporation into engineering correlations and predictive frameworks.

We acknowledge that the DFT calculations represent gas-phase pairwise interactions and inherently neglect condensed-phase many-body effects, including cooperative hydrogen bonding, dielectric screening, and thermal entropy. Consequently, the absolute binding energies should not be directly equated to liquid-phase interaction free energies. However, the DFT results provide critical insights into the relative intrinsic strengths of self-*versus* cross-association and the electronic influence of alkyl chain length, serving as benchmarks for force field validation. The classical MD simulations compensate for these limitations by explicitly accounting for many-body packing, thermal fluctuations, and bulk dielectric environments. Thus, DFT elucidates the quantum mechanical origins of molecular recognition, while MD translates these preferences into statistical thermodynamic behavior, rendering the methodologies complementary in establishing the structure–property relationships reported herein.

## 4. Conclusion

This work presents a multiscale structural investigation of propyl ethanoate + C_6_–C_10_ 1-alkanol mixtures, integrating experimental thermophysical measurements with molecular dynamics simulations and quantum chemical calculations to elucidate the molecular origins of non-ideal mixing behavior. Experimentally, systematic density and viscosity measurements revealed positive excess molar volumes and negative viscosity deviations that increase monotonically with alcohol chain length. These macroscopic trends reflect increasingly inefficient molecular packing and disrupted hydrogen-bond networks, directly attributable to the structural asymmetry between strong alcohol self-association and weak cross-species interactions. Molecular dynamics simulations reveal that alcohol–alcohol self-association dominates through directional hydrogen bonding with dual-donor/acceptor geometry, forming transient hydrogen-bonded clusters. Radial and spatial distribution functions demonstrate robust positional correlations between alcohol molecules, whereas alcohol–ester hydrogen bonding is highly site-specific, occurring exclusively at the ester carbonyl oxygen with markedly weaker intensity. Void space analysis further confirms that this structural hierarchy generates expanded free volume and reduced packing efficiency upon mixing. Quantum chemical calculations and atoms-in-molecules analysis provide quantitative validation: alcohol–alcohol binding energies strengthen significantly with chain length (−8.24 to −11.31 kcal mol^−1^) due to cumulative dispersion interactions between alkyl chains, while alcohol–propyl ethanoate interactions remain nearly invariant (∼−8.30 kcal mol^−1^). AIM topological parameters confirm increasing bond critical points associated with dispersion interactions as chain length increases.

The detailed elucidation of intermolecular interactions provided in this work offers a robust theoretical framework for developing more accurate predictive thermodynamic models. Furthermore, this knowledge is directly applicable to the design, simulation, and optimization of efficient green solvent extraction processes utilizing propyl ethanoate and higher alkanols across various industrial applications.

## Author contributions

Mohammad Almasi: experimental, conceptualization, methodology, writing – original draft. Morteza Vatanparast: investigation, writing – review & editing, data curation. Adel Noubigh: validation, writing – review & editing. All authors reviewed and approved the final manuscript.

## Conflicts of interest

The authors declare no competing interests.

## Supplementary Material

RA-016-D6RA01677D-s001

## Data Availability

All data generated or analyzed during the current study are available within the manuscript and its accompanying supplementary information (SI). Supplementary information: experimental density and viscosity alongside the excess values of thermodynamic parameters at differnt temperatures are reported. See DOI: https://doi.org/10.1039/d6ra01677d.
