# Supplementary material for: Investigation of propyl ethanoate + C6–C10 1-alkanols: experimental properties, molecular dynamics, and quantum chemical insights
Source: RSC Adv. 2026 Apr 29;16(25):22375–85. doi: 10.1039/d6ra01677d (PMC13126651; doi:10.1039/d6ra01677d)
Supplement: RA-016-D6RA01677D-s001 [file RA-016-D6RA01677D-s001.pdf]

Supplementary Information (SI) for RSC Advances.
This journal is © The Royal Society of Chemistry 2026

Investigation of Propyl Ethanoate + C_6-C_{10} 1-Alkanols: Experimental Properties, Molecular Dynamics, and Quantum Chemical Insights

Mohammad Almasi[*1], Morteza Vatanparast[1], Adel Noubigh[2]

[1] Department of Applied Chemistry, Faculty of Science, Malayer University, Malayer, 65174, Iran

[2] Center for Scientific Research and Entrepreneurship, Northern Border University, 73213, Arar, Saudi Arabia

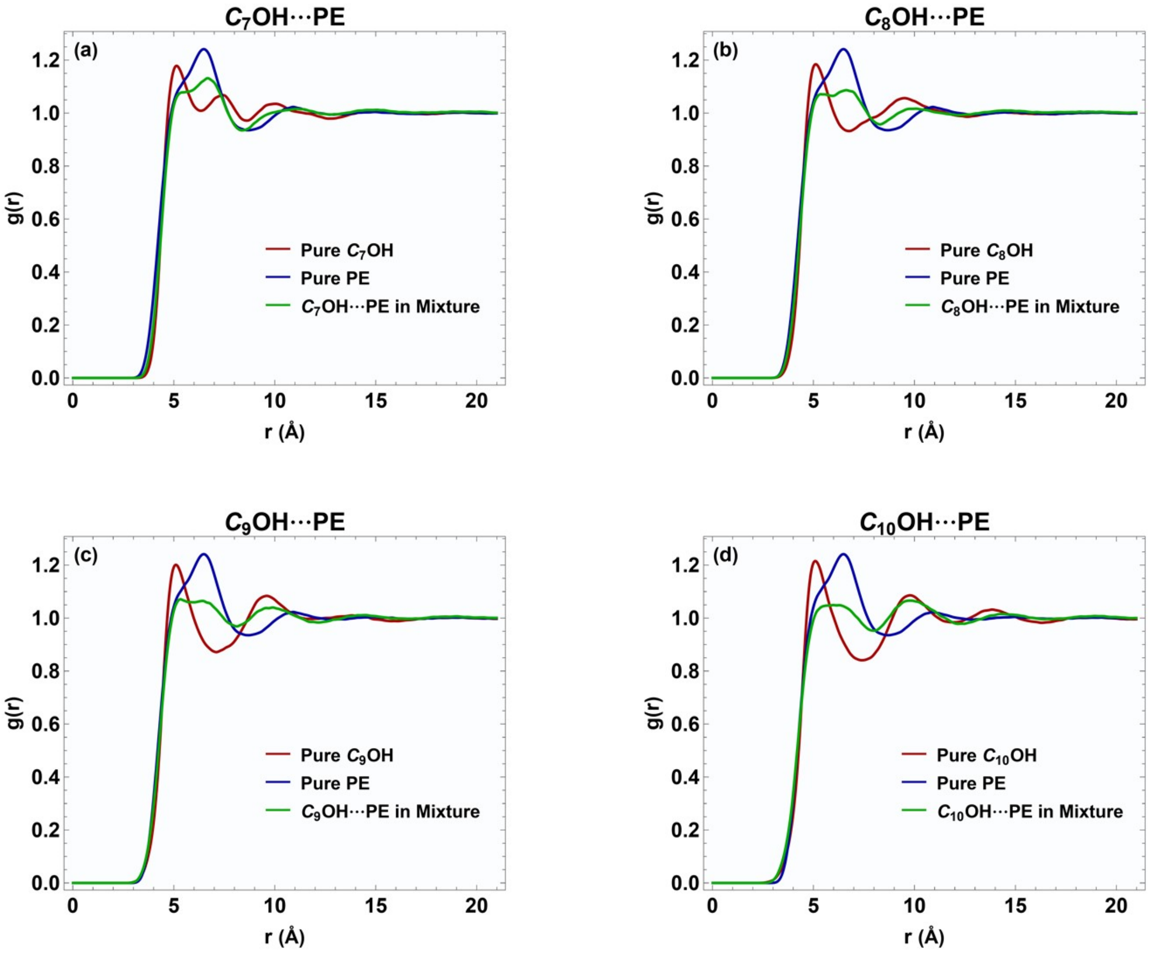

Fig. S1. Center of geometry RDF, g(r), for ROH–PE interactions in equimolar mixtures (x1 = 0.5) at 293.15 K.

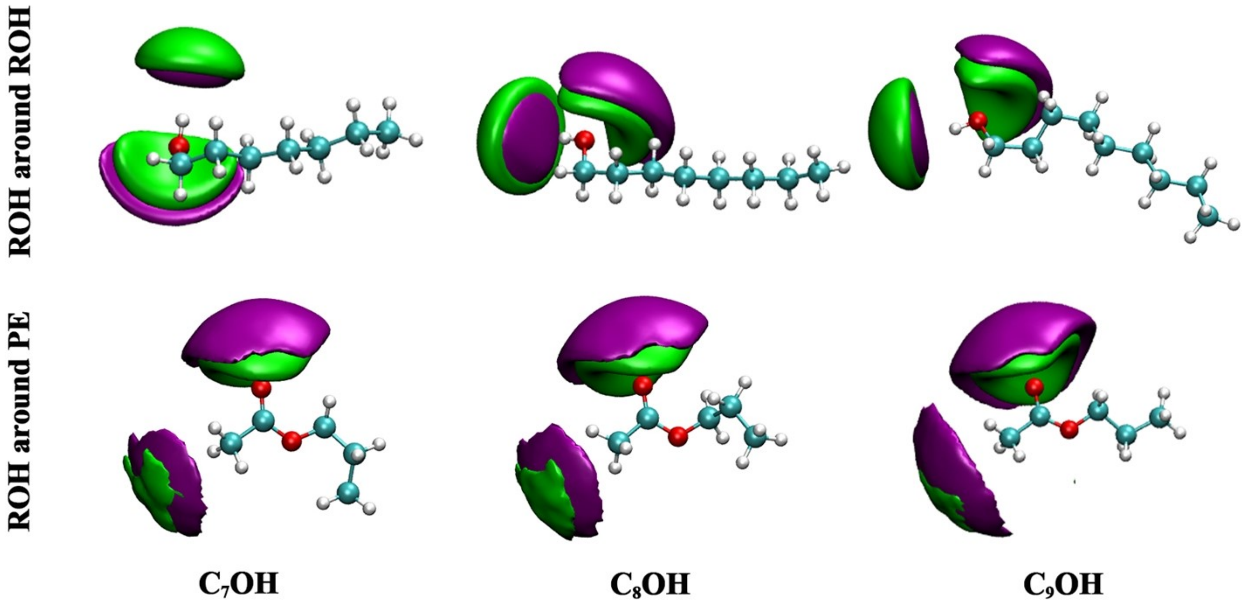

Fig. S2. Spatial distribution functions showing the three-dimensional probability density of ROH around ROH and PE in the equimolar mixture (x1 = 0.5) at 293.15 K, where green lobes correspond to hydrogen atoms and purple regions indicate oxygen atoms.

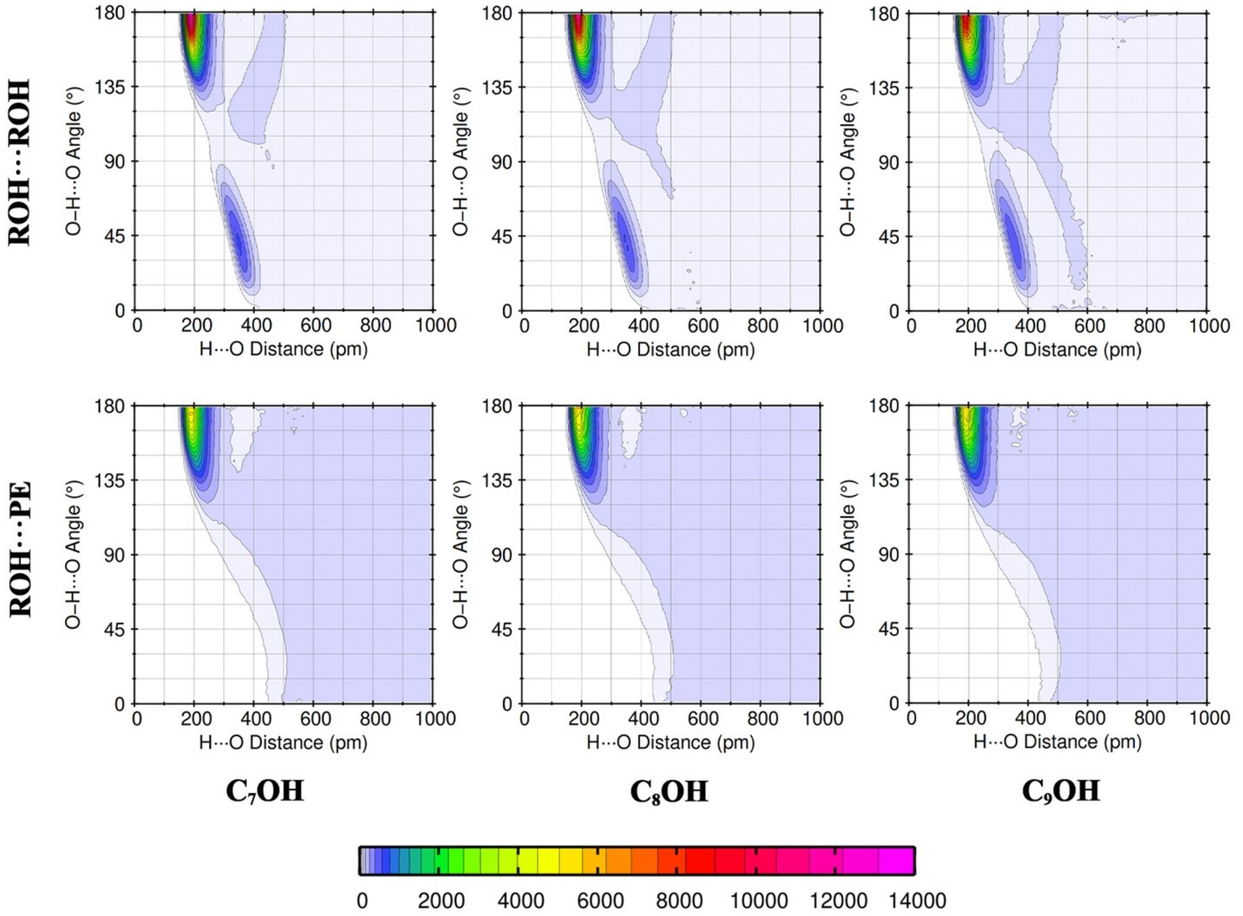

Fig. S3. Computed combined distribution functions involving the O–H···O hydrogen-bond angle and the H···O distance in the equimolar mixture (x1 = 0.5) at 293.15 K.

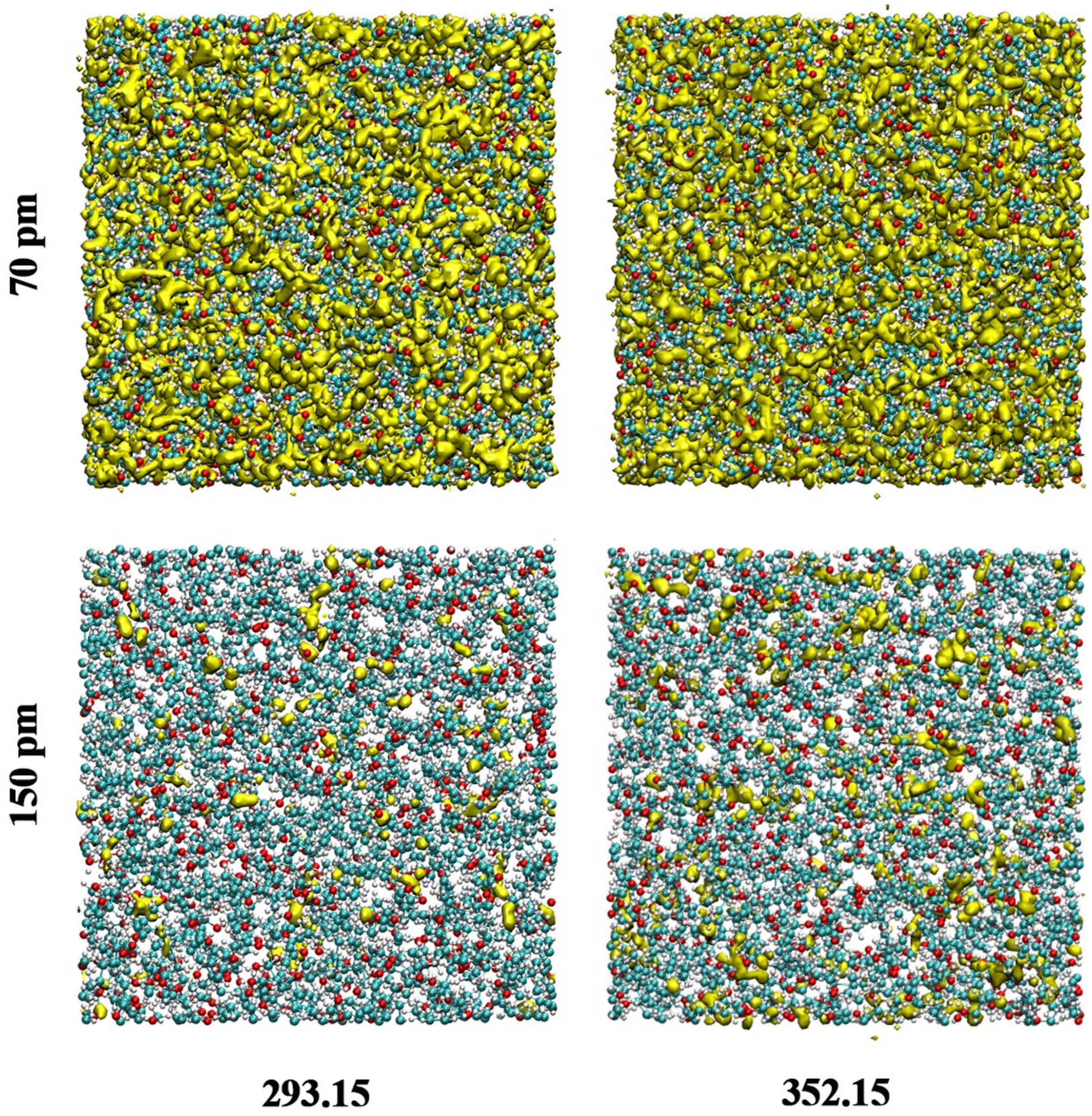

Fig. S4. Simulation snapshots illustrating void spheres with radii of 70 and 150 pm in the PE + C_6OH system (yellow regions) in the equimolar mixture (x_1 = 0.5) at 293.15 K.

Table S1. Experimental density and viscosity of mixtures at different temperatures and pressure 0.1 MPa.

Propyl Ethanoate (1) + 1-Hexanol (2)				
		ρ(g.cm^{-3})		
x_1	T(K) =293.15	T(K) =303.15	T(K) =313.15	T(K) =323.15
0	0.8187	0.8115	0.8042	0.7968
0.0836	0.8235	0.8159	0.8082	0.8004
0.1638	0.8282	0.8202	0.8122	0.8040
0.2557	0.8338	0.8254	0.8170	0.8084
0.3542	0.8401	0.8313	0.8224	0.8134
0.4561	0.8468	0.8376	0.8283	0.8189
0.5536	0.8535	0.8439	0.8342	0.8244
0.6542	0.8608	0.8507	0.8406	0.8304
0.7528	0.8682	0.8577	0.8472	0.8366
0.8557	0.8762	0.8654	0.8545	0.8434
0.9536	0.8842	0.8730	0.8617	0.8502
1	0.8881	0.8767	0.8652	0.8535
		η (mPa.s)		
0	5.37	3.94	2.95	2.26
0.0836	4.554	3.296	2.453	1.918
0.1638	3.922	2.843	2.131	1.686
0.2557	3.368	2.449	1.852	1.479
0.3542	2.886	2.099	1.595	1.288
0.4561	2.438	1.771	1.356	1.108
0.5536	2.050	1.487	1.144	0.949
0.6542	1.677	1.225	0.944	0.798
0.7528	1.344	0.997	0.777	0.668
0.8557	1.004	0.774	0.617	0.539
0.9536	0.712	0.593	0.503	0.448
1	0.587	0.519	0.461	0.409

Propyl Ethanoate (1) + 1-Heptanol (2)				
		ρ(g.cm^{-3})		
x_1	T(K) =293.15	T(K) =303.15	T(K) =313.15	T(K) =323.15
0	0.8222	0.8152	0.8080	0.8007
0.0817	0.8260	0.8187	0.8111	0.8033
0.1619	0.8300	0.8223	0.8144	0.8063
0.2544	0.8349	0.8268	0.8185	0.8101
0.3539	0.8405	0.8320	0.8233	0.8145
0.4528	0.8465	0.8376	0.8285	0.8193
0.5552	0.8531	0.8438	0.8343	0.8247
0.6539	0.8600	0.8502	0.8403	0.8302
0.7543	0.8675	0.8572	0.8468	0.8363
0.8547	0.8755	0.8648	0.8539	0.8429
0.9539	0.8839	0.8728	0.8615	0.8500
1	0.8881	0.8767	0.8652	0.8535
		η (mPa.s)		
0	7.03	5.03	3.71	2.79
0.0817	6.046	4.315	3.165	2.395
0.1619	5.255	3.779	2.786	2.114
0.2544	4.532	3.286	2.436	1.852
0.3539	3.880	2.825	2.102	1.609
0.4528	3.286	2.397	1.794	1.380
0.5552	2.713	1.984	1.492	1.161
0.6539	2.195	1.617	1.219	0.964
0.7543	1.701	1.270	0.969	0.780
0.8547	1.222	0.942	0.736	0.608
0.9539	0.778	0.644	0.540	0.469
1	0.587	0.519	0.461	0.409

Propyl Ethanoate (1) + 1-Octanol (2)				
		ρ(g.cm^{-3})		
x_1	T(K) =293.15	T(K) =303.15	T(K) =313.15	T(K) =323.15
0	0.8249	0.8179	0.8109	0.8037
0.0829	0.8283	0.8209	0.8136	0.8061
0.1603	0.8316	0.8239	0.8163	0.8085
0.2512	0.8358	0.8278	0.8199	0.8117
0.3518	0.8409	0.8325	0.8242	0.8156
0.4524	0.8465	0.8377	0.8289	0.8199
0.5533	0.8527	0.8435	0.8342	0.8247
0.6541	0.8594	0.8498	0.8401	0.8301
0.7519	0.8666	0.8565	0.8463	0.8359
0.8536	0.8748	0.8642	0.8535	0.8426
0.9545	0.8838	0.8726	0.8614	0.8499
1	0.8881	0.8767	0.8652	0.8535
		η (mPa.s)		
0	9.13	6.41	4.63	3.40
0.0829	7.923	5.545	3.980	2.934
0.1603	6.963	4.900	3.528	2.604
0.2512	6.049	4.281	3.096	2.289
0.3518	5.177	3.674	2.664	1.979
0.4524	4.365	3.103	2.261	1.687
0.5533	3.597	2.563	1.871	1.411
0.6541	2.865	2.053	1.504	1.151
0.7519	2.197	1.593	1.179	0.918
0.8536	1.510	1.129	0.857	0.687
0.9545	0.852	0.687	0.564	0.481
1	0.544	0.484	0.433	0.386

Propyl Ethanoate (1) + 1-Nonanol (2)				
		ρ(g.cm^{-3})		
x_1	T(K) =293.15	T(K) =303.15	T(K) =313.15	T(K) =323.15
0	0.8275	0.8205	0.8136	0.8065
0.0824	0.8303	0.8230	0.8158	0.8084
0.1619	0.8333	0.8257	0.8182	0.8105
0.2531	0.8370	0.8291	0.8213	0.8132
0.3534	0.8416	0.8333	0.8251	0.8167
0.4559	0.8469	0.8382	0.8296	0.8208
0.5527	0.8525	0.8434	0.8343	0.8251
0.6552	0.8591	0.8495	0.8399	0.8302
0.7549	0.8663	0.8562	0.8461	0.8359
0.8538	0.8743	0.8638	0.8532	0.8424
0.9544	0.8835	0.8724	0.8612	0.8498
1	0.8881	0.8767	0.8652	0.8535
		η (mPa.s)		
0	11.41	7.86	5.59	4.10
0.0824	9.977	6.846	4.837	3.559
0.1619	8.793	6.057	4.290	3.158
0.2531	7.657	5.298	3.764	2.775
0.3534	6.557	4.547	3.236	2.396
0.4559	5.500	3.816	2.727	2.026
0.5527	4.553	3.164	2.265	1.698
0.6552	3.584	2.504	1.797	1.364
0.7549	2.686	1.897	1.376	1.062
0.8538	1.813	1.318	0.978	0.776
0.9544	0.945	0.750	0.606	0.511
1	0.544	0.484	0.433	0.386

Propyl Ethanoate (1) + 1-Decanol (2)				
		ρ(g.cm^{-3})		
x_1	T(K) =293.15	T(K) =303.15	T(K) =313.15	T(K) =323.15
0	0.8295	0.8227	0.8158	0.8089
0.0842	0.8320	0.8249	0.8178	0.8106
0.1651	0.8346	0.8273	0.8199	0.8124
0.2533	0.8378	0.8302	0.8225	0.8147
0.3548	0.8421	0.8341	0.8260	0.8178
0.4536	0.8469	0.8384	0.8299	0.8213
0.5529	0.8523	0.8434	0.8344	0.8254
0.6547	0.8586	0.8492	0.8397	0.8302
0.7535	0.8656	0.8557	0.8457	0.8356
0.8552	0.8740	0.8634	0.8529	0.8422
0.9558	0.8835	0.8723	0.8611	0.8497
1	0.8881	0.8767	0.8652	0.8535
		η (mPa.s)		
0	14.42	9.76	6.84	4.96
0.0842	12.663	8.535	5.942	4.318
0.1651	11.191	7.562	5.274	3.834
0.2533	9.807	6.649	4.647	3.380
0.3548	8.389	5.695	3.985	2.908
0.4536	7.078	4.807	3.373	2.469
0.5529	5.816	3.953	2.778	2.049
0.6547	4.561	3.113	2.192	1.632
0.7535	3.392	2.337	1.659	1.255
0.8552	2.201	1.558	1.130	0.879
0.9558	1.056	0.816	0.648	0.537
1	0.544	0.484	0.433	0.386

Table S2. Excess molar volumes and viscosity deviations of mixtures at different temperatures.

Propyl Ethanoate (1) + 1-Hexanol (2)				
$V_m^E (cm^3.mol^{-1})$				
x_1	T(K) =293.15	T(K) =303.15	T(K) =313.15	T(K) =323.15
0.0836	0.088	0.104	0.120	0.135
0.1638	0.165	0.198	0.215	0.247
0.2557	0.233	0.273	0.299	0.338
0.3542	0.275	0.317	0.360	0.400
0.4561	0.306	0.347	0.388	0.424
0.5536	0.310	0.349	0.389	0.422
0.6542	0.276	0.323	0.358	0.384
0.7528	0.226	0.266	0.294	0.312
0.8557	0.155	0.170	0.186	0.204
0.9536	0.055	0.059	0.064	0.069
$\Delta\eta$ (mPa.s)				
0.0836	-0.416	-0.358	-0.289	-0.187
0.1638	-0.665	-0.537	-0.411	-0.271
0.2557	-0.779	-0.616	-0.462	-0.308
0.3542	-0.790	-0.629	-0.473	-0.316
0.4561	-0.750	-0.609	-0.459	-0.308
0.5536	-0.672	-0.559	-0.428	-0.286
0.6542	-0.564	-0.477	-0.378	-0.251
0.7528	-0.425	-0.368	-0.299	-0.199
0.8557	-0.273	-0.239	-0.203	-0.137
0.9536	-0.097	-0.085	-0.073	-0.047

Propyl Ethanoate (1) + 1-Heptanol (2)				
		$V_m^E (cm^3.mol^{-1})$		
x_1	T(K) =293.15	T(K) =303.15	T(K) =313.15	T(K) =323.15
0.0817	0.106	0.111	0.136	0.177
0.1619	0.184	0.210	0.239	0.284
0.2544	0.252	0.289	0.330	0.368
0.3539	0.305	0.344	0.389	0.430
0.4528	0.326	0.364	0.409	0.448
0.5552	0.329	0.360	0.399	0.431
0.6539	0.295	0.332	0.363	0.401
0.7543	0.236	0.273	0.306	0.330
0.8547	0.157	0.175	0.205	0.224
0.9539	0.064	0.060	0.069	0.080
		$\Delta\eta$ (mPa.s)		
0.0817	-0.458	-0.346	-0.280	-0.200
0.1619	-0.732	-0.521	-0.398	-0.291
0.2544	-0.859	-0.596	-0.447	-0.332
0.3539	-0.870	-0.609	-0.458	-0.338
0.4528	-0.827	-0.590	-0.445	-0.332
0.5552	-0.740	-0.541	-0.414	-0.307
0.6539	-0.622	-0.463	-0.366	-0.269
0.7543	-0.469	-0.357	-0.290	-0.214
0.8547	-0.301	-0.232	-0.197	-0.147
0.9539	-0.106	-0.083	-0.071	-0.050

Propyl Ethanoate (1) + 1-Octanol (2)				
		$V_m^E (cm^3.mol^{-1})$		
x_1	T(K) =293.15	T(K) =303.15	T(K) =313.15	T(K) =323.15
0.0829	0.094	0.123	0.132	0.141
0.1603	0.184	0.223	0.241	0.260
0.2512	0.266	0.303	0.318	0.352
0.3518	0.324	0.366	0.386	0.424
0.4524	0.351	0.393	0.429	0.465
0.5533	0.342	0.378	0.423	0.469
0.6541	0.320	0.345	0.376	0.424
0.7519	0.260	0.285	0.315	0.346
0.8536	0.174	0.189	0.208	0.226
0.9545	0.055	0.068	0.069	0.083
		$\Delta\eta$ (mPa.s)		
0.0829	-0.499	-0.377	-0.304	-0.218
0.1603	-0.798	-0.566	-0.434	-0.317
0.2512	-0.935	-0.649	-0.487	-0.360
0.3518	-0.948	-0.664	-0.499	-0.369
0.4524	-0.900	-0.642	-0.483	-0.36
0.5533	-0.806	-0.588	-0.452	-0.334
0.6541	-0.677	-0.504	-0.399	-0.293
0.7519	-0.510	-0.388	-0.316	-0.233
0.8536	-0.328	-0.252	-0.214	-0.160
0.9545	-0.124	-0.100	-0.087	-0.064

Propyl Ethanoate (1) + 1-Nonanol (2)				
		$V_m^E (cm^3.mol^{-1})$		
x_1	T(K) =293.15	T(K) =303.15	T(K) =313.15	T(K) =323.15
0.0824	0.120	0.136	0.150	0.164
0.1619	0.207	0.237	0.262	0.288
0.2531	0.299	0.331	0.355	0.400
0.3534	0.357	0.398	0.429	0.461
0.4559	0.378	0.419	0.449	0.478
0.5527	0.367	0.407	0.449	0.473
0.6552	0.332	0.374	0.415	0.440
0.7549	0.271	0.309	0.342	0.360
0.8538	0.185	0.197	0.214	0.232
0.9544	0.068	0.071	0.076	0.082
		$\Delta\eta$ (mPa.s)		
0.0824	-0.541	-0.409	-0.330	-0.237
0.1619	-0.865	-0.614	-0.470	-0.344
0.2531	-1.014	-0.704	-0.528	-0.391
0.3534	-1.028	-0.719	-0.541	-0.400
0.4559	-0.976	-0.697	-0.525	-0.391
0.5527	-0.875	-0.639	-0.490	-0.362
0.6552	-0.735	-0.546	-0.432	-0.318
0.7549	-0.554	-0.421	-0.342	-0.252
0.8538	-0.356	-0.274	-0.233	-0.173
0.9544	-0.136	-0.104	-0.089	-0.066

Propyl Ethanoate (1) + 1-Decanol (2)				
$V_m^E (cm^3.mol^{-1})$				
x_1	T(K) =293.15	T(K) =303.15	T(K) =313.15	T(K) =323.15
0.0842	0.128	0.146	0.142	0.157
0.1651	0.244	0.255	0.267	0.296
0.2533	0.337	0.355	0.372	0.403
0.3548	0.387	0.414	0.442	0.479
0.4536	0.396	0.445	0.476	0.514
0.5529	0.389	0.432	0.475	0.504
0.6547	0.354	0.397	0.440	0.465
0.7535	0.291	0.325	0.360	0.390
0.8552	0.180	0.221	0.234	0.253
0.9558	0.057	0.078	0.086	0.096
$\Delta\eta$ (mPa.s)				
0.0842	-0.592	-0.447	-0.361	-0.259
0.1651	-0.945	-0.672	-0.513	-0.375
0.2533	-1.109	-0.770	-0.577	-0.427
0.3548	-1.123	-0.786	-0.592	-0.437
0.4536	-1.067	-0.761	-0.573	-0.427
0.5529	-0.956	-0.698	-0.535	-0.395
0.6547	-0.803	-0.597	-0.472	-0.348
0.7535	-0.605	-0.460	-0.374	-0.276
0.8552	-0.389	-0.299	-0.255	-0.189
0.9558	-0.142	-0.111	-0.095	-0.073

x_1 is the mole fraction of Propyl ethanoate in binary mixtures. Standard uncertainties u are $u(T)$ = 0.03 K, $u(\rho)$ = 0.0005 g.cm^{-3}, $u(V_m^E)$ = 0.006 cm^3.mol^{-1}, $u(\Delta\eta)$ = 0.009 mPa.s, $u_r(p)$ = 0.01, $u_r(\eta)$ = 0.05
